# Supplementary material for: Prolonged bacterial lag time results in small colony variants that represent a sub-population of persisters
Source: Nat Commun. 2018 Oct 4;9:4074. doi: 10.1038/s41467-018-06527-0 (PMC6172231; doi:10.1038/s41467-018-06527-0)
Supplement: Supplementary file 1 — Supplementary Information [file 41467_2018_6527_MOESM1_ESM.pdf]

# Supplementary material for:

# Prolonged bacterial lag time results in small colony variants that represent a sub-population of persisters

Vulin, Leimer, Huemer et al.

**Contents**

1) Agar pad protocol
2) Analyzing growth rates of colonies with different lag times
3) Testing speed of reversion to wild type colony size
4) Colony growth model: estimation of lag time from 24h colony size
5) Survival of pre-exposed bacteria upon antibiotics exposure
6) Additional data from murine abscess model
7) Gentamicin-induced SCV
8) Descriptors of colony size distributions
9) Growth rate of bacteria in acidic conditions
10) Repartition of growth events and lysis events
11) Description of supplementary movie

**Supplementary Figure 1: Agar pad method used for long-term observation of bacteria using time-lapse microscopy**

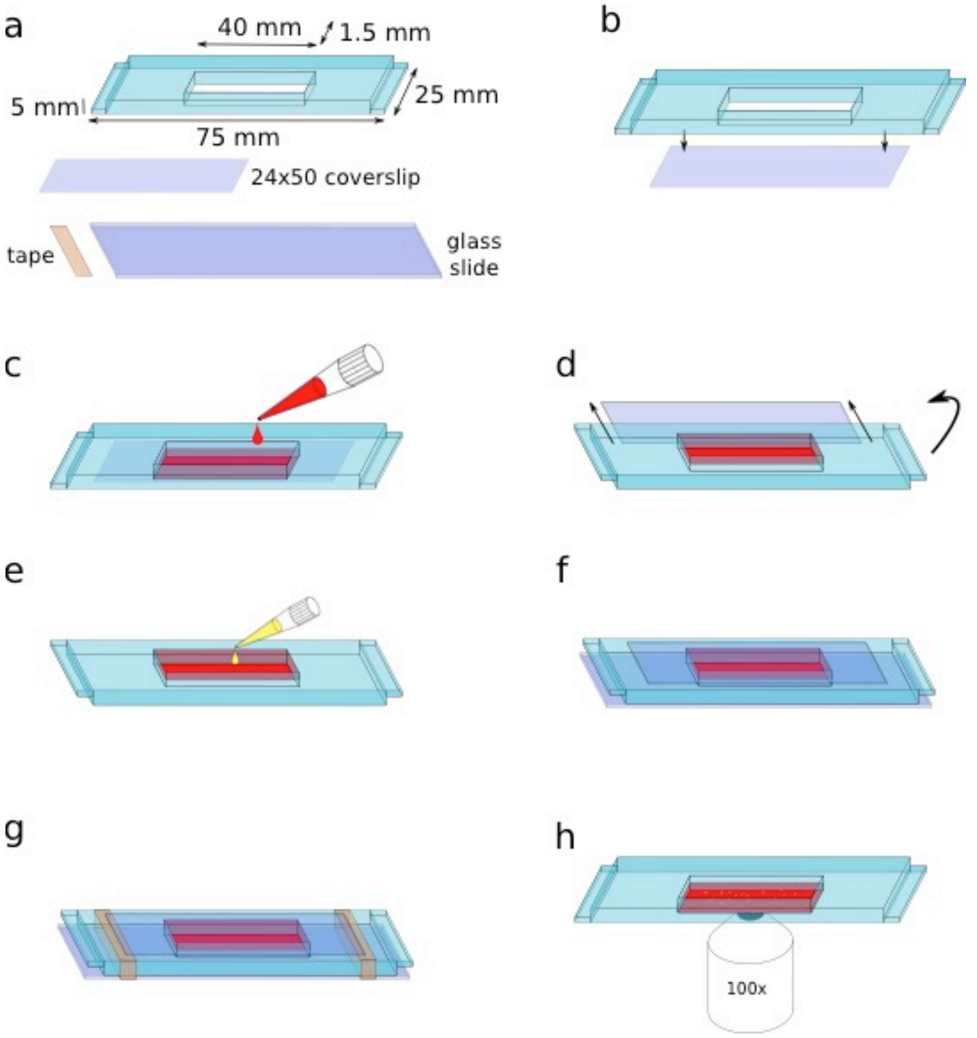


**Pad preparation**

b) A custom frame made out of polyvinylchloride (see panel A for dimensions) was placed on top of a 25x50 mm coverslip.
c) 1.9 ml of melted 40°C agar media was casted into the frame and left to solidify (1-5 minutes).

**Inoculation**

d) The device was then covered with a glass slide and flipped upside down. The coverslip was gently slided outwards to uncover the agar surface.
e) 8 µl of bacterial suspension was pipetted onto the agar pad and distributed using a pipet tip until liquid was absorbed.
f) The coverslip was replaced to seal the system.
g) The system was sealed using office tape.

**Supplementary Figure 2: Analyzing growth rates of colonies with different lag times**

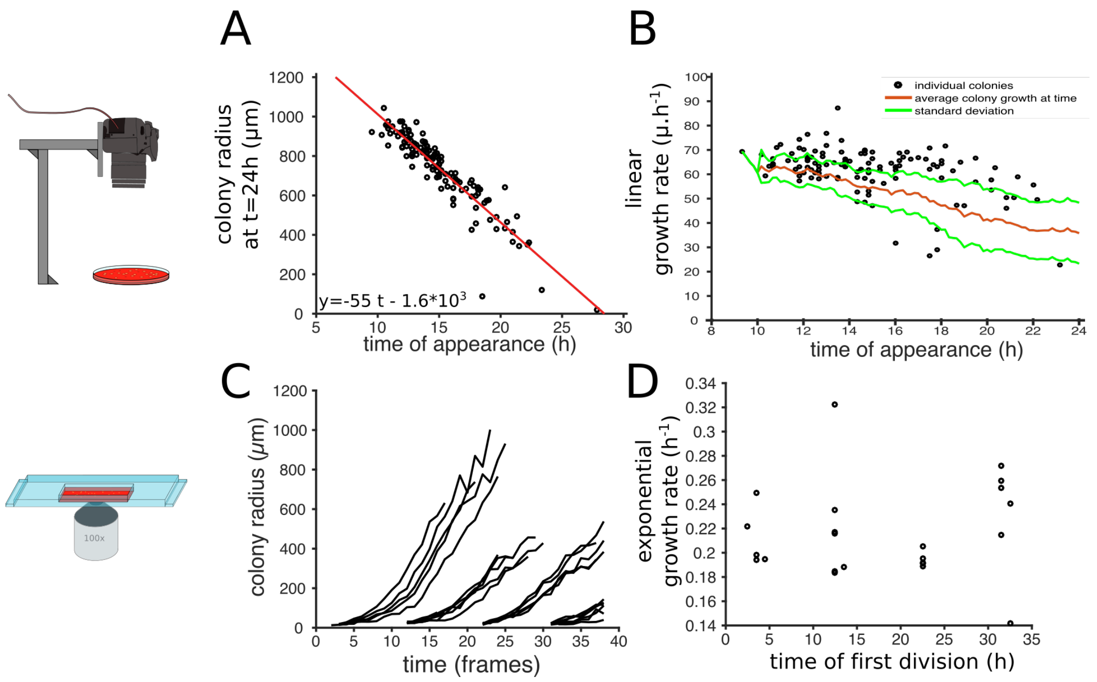


At millimeter scale (**top**), appearance time of the colonies (the time when a colony reached a radius of 80 µm) was predictive of the colony radius at 24h ($R^2$=0.83) (**A**). The initial growth rate of colonies (**B**) was slowly decreasing as a function of their appearance time as expected because of nutrients depletion on a plate. The rate at which a given colony grew also decreased over time (red line; green lines are 95% confidence intervals). These observations were supported by microscopic analysis (**bottom**): the colony radius initially grew exponentially (**C**). The initial exponential growth rate of colonies (**D**) did not correlate with the time of first division.

**Supplementary Figure 3: Testing speed of reversion to wild type colony size**

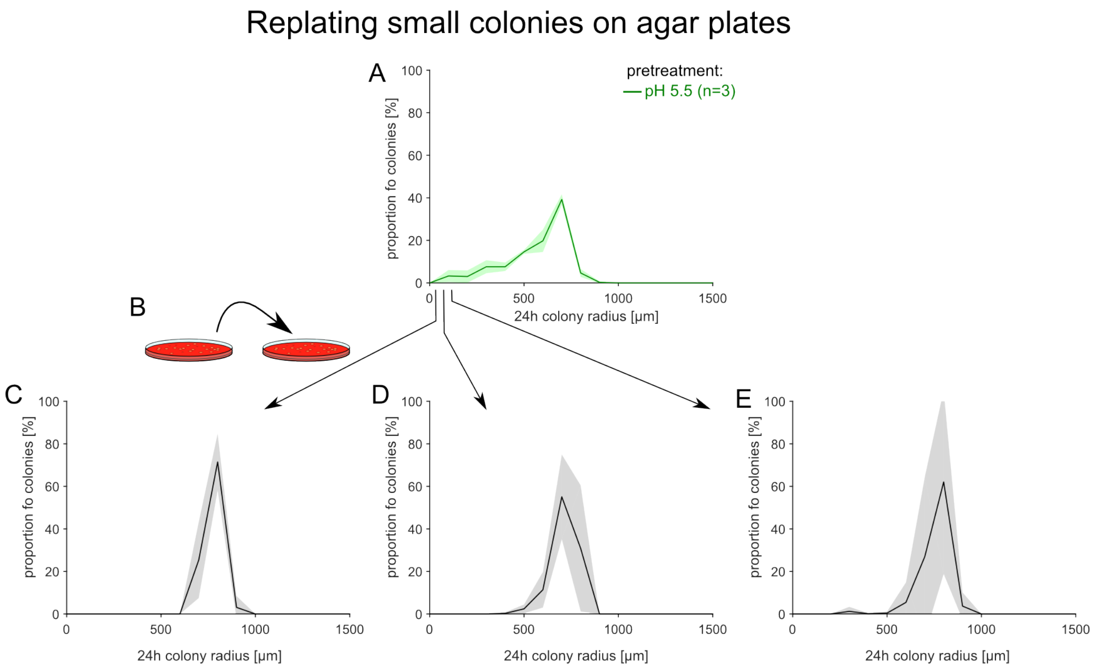


Small colonies, obtained from bacteria grown in DMEM pH 5.5 for 3 days, sampled 24 hours after plating from the tail of a wide distribution (**A**) will restore a wild type colony size distribution if plated immediately (**B**). The distributions that are obtained are peaked with no small colony variants, similarly to the distribution obtained by sampling bacteria from an exponentially growing culture. **C, D, E** are mean distributions from three replated SCVs; each represents one biological replicate. This shows that in addition to the restoration of the growth rate phenotype immediately after first divisions (supplementary material 2), the width of colony size distribution is also restored upon sub-cultivation.

**Supplementary Figure 4: Colony growth model: estimation of lag time from 24h colony size**

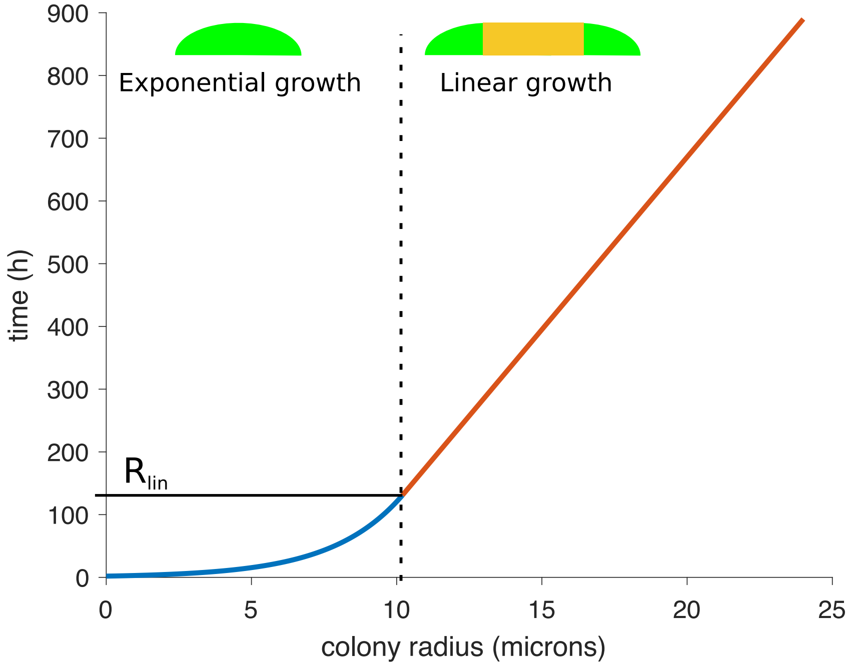


One can estimate initial lag time of the bacterium that initiated a colony by extrapolation based on the colony radius at 24 h. For this, we assumed a two-step growth dynamics. First, the colony grew exponentially at a rate of 0.41 $h^{-1}$ (blue line, as observed under the microscope) until the radius reached $R_{lin}$=130 µm. Once the colony has reached this radius, cells in the center cannot access nutrients anymore, and only a fixed band of bacteria at the colony edge will be dividing, with the consequence that radial growth is linear (39) (orange line). We fitted this growth rate on time lapse movies from 3 different plates to be on average 55 µm/h. We use this model to estimate lag time from colony size data.

Note that two problems emerge for the "size ratio" commonly used to define SCVs. First, this definition is based on using the most common colony size as the denominator, which will change with SCV proportion (see **Fig. 1D** for example). As a consequence, the SCV proportion can be underestimated. Second, since the growth of colonies is linear, it results that the ratio of sizes between "large" colonies and small colonies is time dependent: the ratio between the radii of two colonies will tend towards 1 for infinite time (in linear growth). Below a comparison of two colonies with different lag times:

| **Time of observation** | **15h** | **20h** | **24h** | **30h** |
|---|---|---|---|---|
| **Radius (µm), Tlag=0 h** | 394 | 669 | 889 | 1219 |
| **Radius (µm), Tlag=6 h** | 80 | 339 | 559 | 889 |
| **Size ratio (radius)** | 4.9 | 2 | 1.6 | 1.4 |
| **Size ratio (area)** | 24 | 3.9 | 2.5 | 1.8 |

**Supplementary Figure 5: Survival of pre-exposed bacteria upon antibiotics exposure**

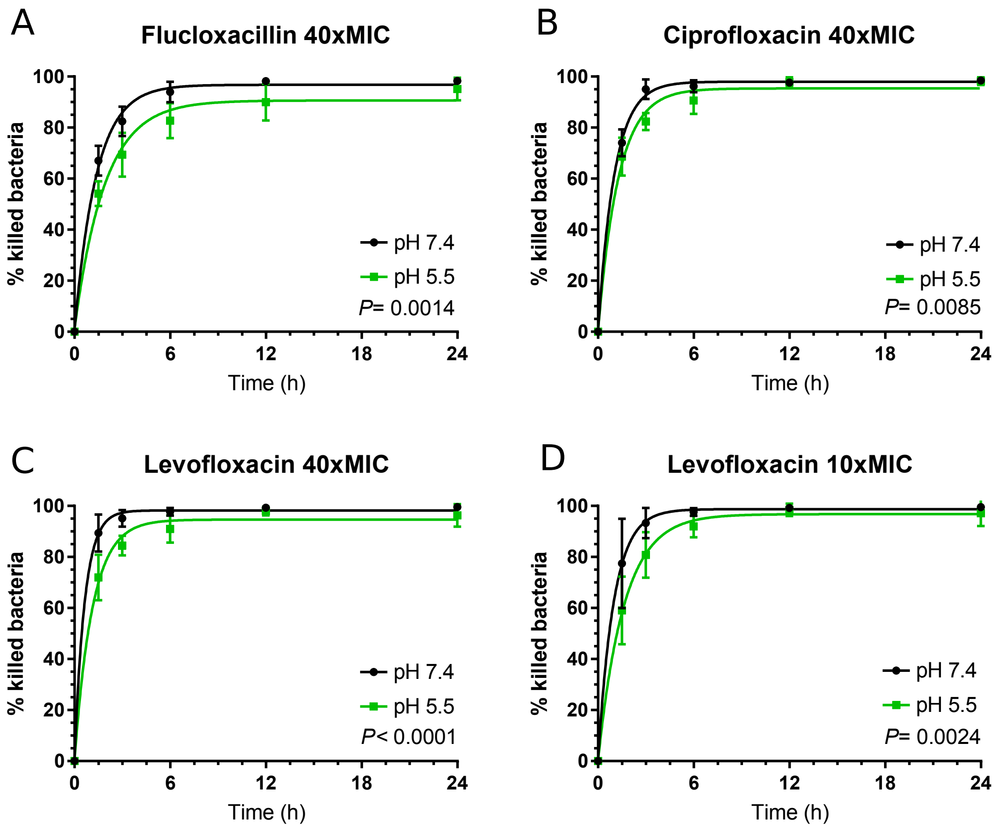


**Time kill curves after pre-exposure to acidic or neutral media.** The curves depict the fraction of bacteria that were killed as a function of time upon exposure to the antibiotics flucloxacillin **(A)** and ciprofloxacin **(B)** at 40 fold the minimum inhibitory concentration. **C** and **D** depict the data for the antibiotic levofloxacin at 40 times and 10 times MIC, respectively. Bacteria were sampled from cultures grown in neutral (pH 7.4) or acidic (pH 5.5) medium for 3 days and transferred to medium at pH 7.4 containing antibiotics. Bacterial survival was analyzed by washing and plating at different time points. Each data point was calculated as the ratio between the number of colony forming units (CFU) at a specific time point after antibiotic addition and the number of CFU in the inoculum. The *P* value at the bottom right of each figure is the result of a one-phase association fit to the time kill data points followed by an extra sum of squares *F* test to compare Y0 of the two fitted curves, their plateau (i.e., the percentage of killed bacteria as time approaches infinity) and *K* values (rate constant, expressed in reciprocal of the X axis time units).

## Supplementary Figure 6: Additional data from murine abscess model

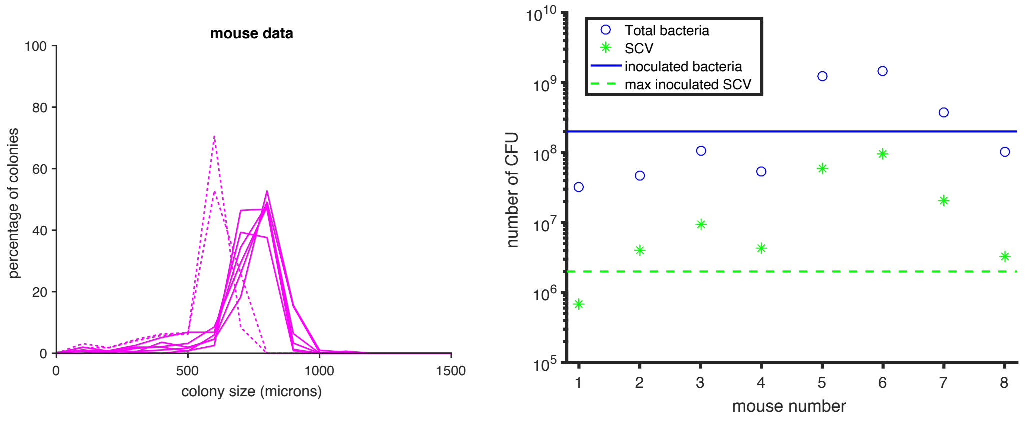


**Left**: Colony sizes distribution after plating samples from 8 mice. Two mouse samples (dashed lines) gave shifted distributions and were thus excluded from the analysis in **Fig. 1**. **Right**: Estimates of the total number of bacterial CFU recovered from mouse samples (blue circles) and the number of SCVs recovered (green stars). The bacteria were sampled from both pus and tissue material surrounding the abscess, whose volume was estimated based on its weight. The horizontal blue line represents the initial inoculum and the green line represents the maximal initial number of inoculated SCVs, as per our detection limit. The samples from mouse 3 and 4 resulted in the shifted distributions (dashed lines) on the left panel.

**Supplementary Figure 7: Gentamicin-induced SCVs**

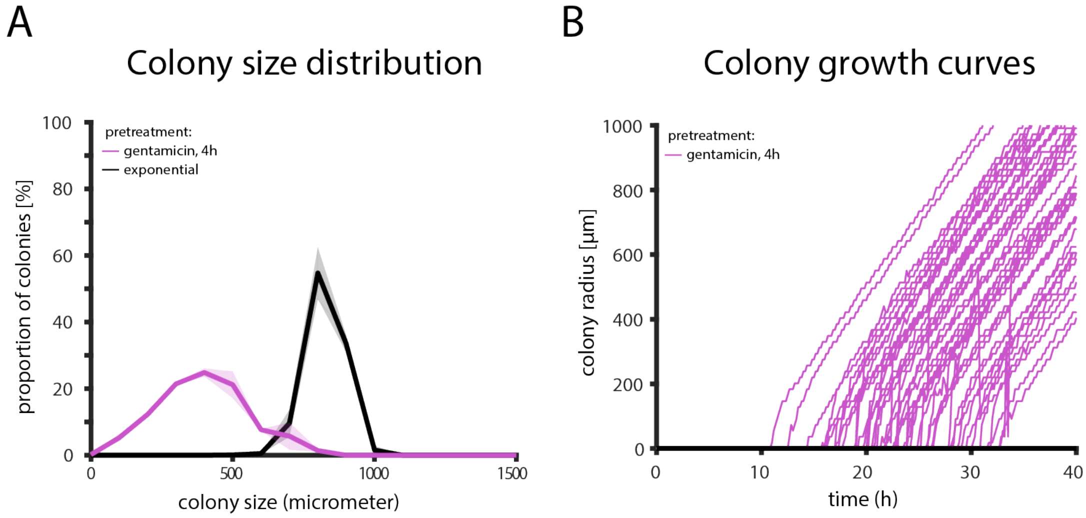


Bacteria treated for 4h in sub-inhibitory concentration of gentamicin (0.25 µg ml$^{-1}$ corresponding to 0.5x MIC) form small colonies. Stationary bacteria from a plate were inoculated in DMEM pH 7.4 to an $OD_{600}$ of 0.2 and incubated at 37°C and 5% $CO_2$ for 4 h. To analyze SCV formation, bacteria were washed twice in PBS and plated. The colony size distribution is wider than that of non-treated bacteria, and most colonies are smaller than bacteria harvested from exponential growth conditions (**A**). When growth resumed, gentamicin-induced small colonies have a similar growth curve as the larger colonies (see **Fig. 2B, C**), with comparable linear growth rates (**B**).

## Supplementary Figure 8: Descriptors of colony size distributions

| | all colonies | | | | colonies >600µm | | | | |
|---|---|---|---|---|---|---|---|---|---|
| cond. | mean | std | all rep. | N col | mean | std | all rep. | N col | % |
| 'Exp1' | 900 | 94 | 848 | 219 | 900 | 94 | 848 | 219 | 100 |
| 'Exp2' | 820 | 51 | | 442 | 820 | 49 | | 441 | 100 |
| 'Exp3' | 824 | 61 | | 329 | 824 | 61 | | 329 | 100 |
| 'pH7_1' | 822 | 72 | 810 | 1901 | 828 | 54 | 802 | 1873 | 99 |
| 'pH7_2' | 787 | 104 | | 92 | 807 | 61 | | 87 | 95 |
| 'pH7_3' | 820 | 84 | | 140 | 829 | 57 | | 137 | 98 |
| 'SCM1' | 731 | 100 | 727 | 239 | 747 | 52 | 761 | 228 | 95 |
| 'SCM2' | 682 | 131 | | 117 | 736 | 56 | | 95 | 81 |
| 'SCM5' | 721 | 126 | | 187 | 756 | 68 | | 166 | 89 |
| 'SCM6' | 716 | 145 | | 151 | 753 | 66 | | 137 | 91 |
| 'SCM7' | 758 | 141 | | 307 | 796 | 73 | | 275 | 90 |
| 'SCM8' | 755 | 125 | | 478 | 780 | 74 | | 447 | 94 |
| 'pH6_1' | 708 | 157 | 696 | 1570 | 772 | 57 | 742 | 1291 | 82 |
| 'pH6_2' | 702 | 162 | | 515 | 770 | 63 | | 420 | 82 |
| 'pH6_3' | 679 | 179 | | 481 | 774 | 68 | | 353 | 73 |
| 'pH5_1' | 650 | 188 | 705 | 1091 | 754 | 49 | 739 | 798 | 73 |
| 'pH5_2' | 731 | 204 | | 84 | 819 | 90 | | 67 | 80 |
| 'pH5_3' | 734 | 199 | | 73 | 832 | 68 | | 56 | 77 |
| 'IC1' | 659 | 163 | 656 | 2709 | 744 | 65 | 772 | 1966 | 73 |
| 'IC2' | 634 | 163 | | 2719 | 735 | 61 | | 1778 | 65 |
| 'IC3' | 676 | 148 | | 2587 | 748 | 65 | | 1940 | 75 |
| 'pH4_1' | 577 | 205 | 543 | 1471 | 735 | 65 | 672 | 833 | 57 |
| 'pH4_3' | 543 | 208 | | 62 | 740 | 73 | | 27 | 44 |
| 'pH4_4' | 508 | 223 | | 622 | 743 | 79 | | 238 | 38 |
| 'Gen1' | 421 | 166 | 403 | 262 | 674 | 66 | 821 | 46 | 18 |
| 'Gen2' | 397 | 166 | | 267 | 678 | 55 | | 38 | 14 |
| 'Gen3' | 392 | 147 | | 175 | 663 | 71 | | 17 | 10 |

The table lists the mean colony sizes observed after 24 hours of growth for each of the replicates in figure 1 and supplementary material 6. The left column contains data for all colonies; the right column contains data for colonies with radius above 600 µm. Standard deviations vary around ~65 µm, which corresponds to approximately 1 hour of growth (55 µm $h^{-1}$ linear growth rate). Of note, pre-exposure to acidic media not only affected the proportion of small colony variants, but to a smaller degree also the mean colony size; this difference in mean size between exponentially growing bacteria and pH 4.0 pre-exposed bacteria is equal to approximately 150 µm, corresponding to about 3 h of growth.

Samples from subcutaneous murine abscess ('SCM') have similar colony sizes as bacteria pre-exposure to media of a pH of 6.5 to 7.4. Samples from intracellular ('IC') experiments have similar colony sizes as bacteria pre-exposure to media of a pH 4.0 to 5.5. The graph below depicts the same data. Error bars represent the standard error of the mean. Data was ordered by pH, and other conditions (SCM, IC and Gen) were ordered according to the mean colony size for larger colonies.

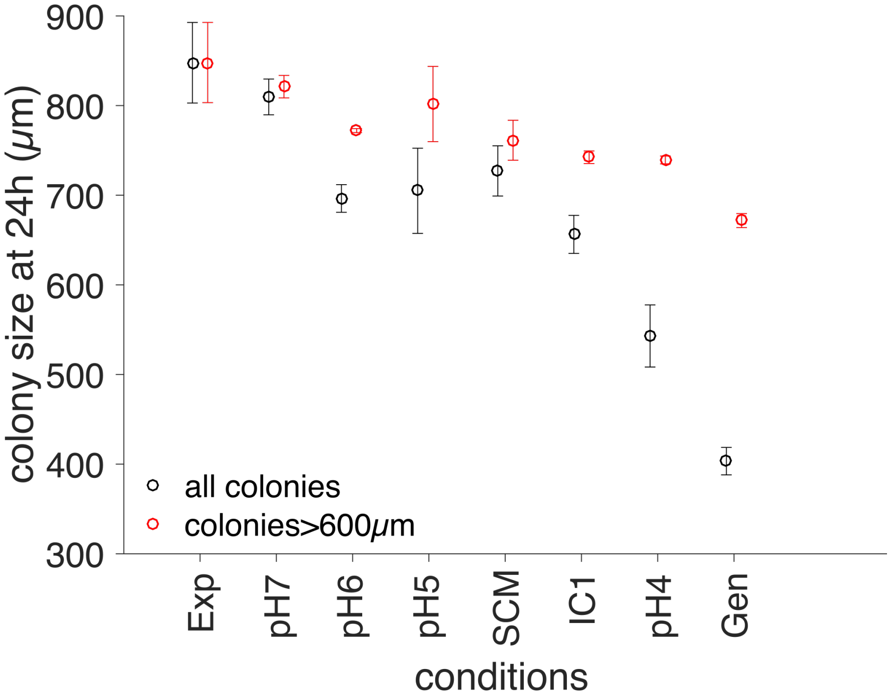

**Supplementary Figure 9: Growth curve of bacteria in acidic and neutral conditions**

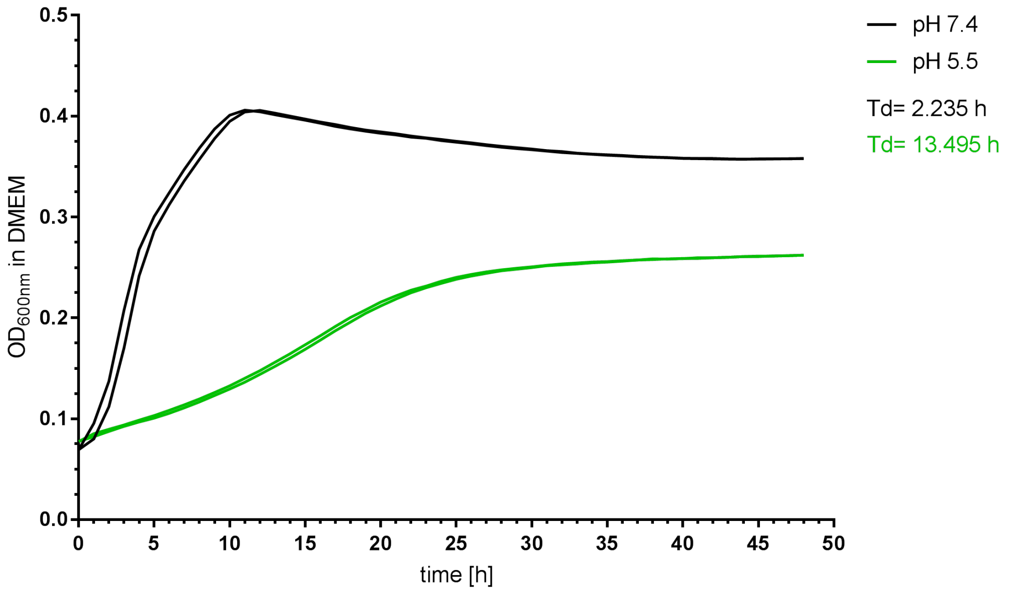


$OD_{600}$ of *S. aureus* strain Cowan was measured over time in pH 5.5 and pH 7.4 medium. Exponential growth rate was fitted between 0 and 5 hours for pH 7.4 incubation, and between 0 and 17 hours for pH 5.5 incubation. We observed growth rates of 0.31 and 0.051 for pH 7.4 and pH 5.5, respectively, which corresponds to doubling times of 2.2 h and 13.5 h.

**Supplementary Figure 10: repartition of growth events and lysis events**

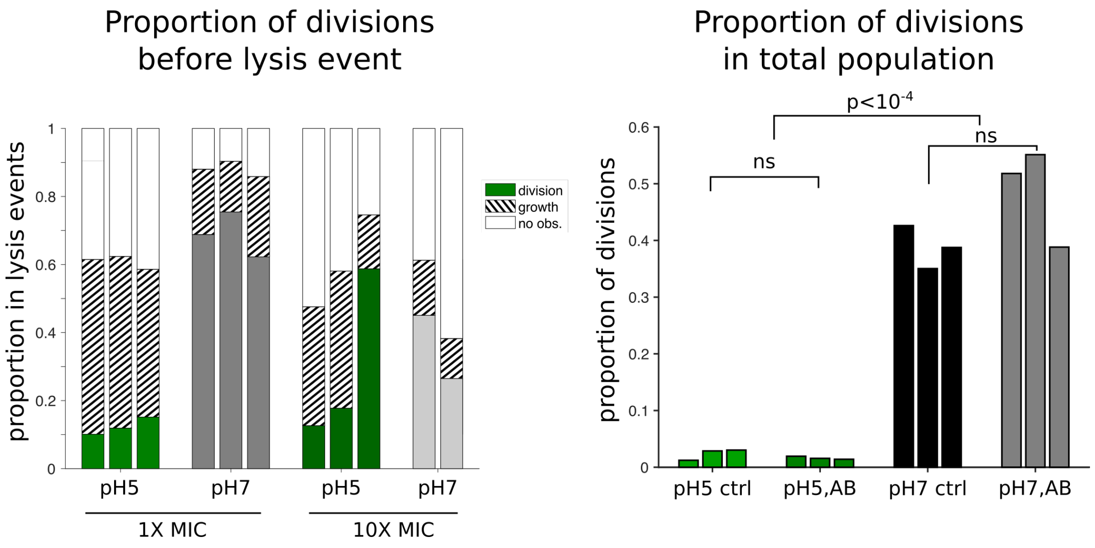


**Left:** When pre-exposed cells were placed on agar pad with flucloxacillin at 1x or 10x MIC, some of them managed to grow (dashed rectangle) or managed at least one division before the lysis occurred (colored rectangle). Note that for many bacteria, no lysis event was observed. **Right**: The total number of cells that managed to divide was sensibly the same whether the agar pad contained antibiotics (1x MIC) or not. The number of cells that did not resume growth at pH 5.5 was lower in pH 5.5 exposed bacteria compared to pH 7.4 exposed bacteria. This matches the data in figure 3D were cells died after saturation.

## Description of supplementary movie 1

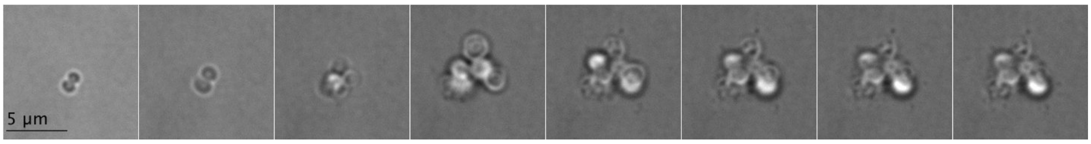


The 8 frames show bacteria regrowing before lysing in presence of antibiotics (flucloxacillin, 0.25 $mgl^{-1}$) after preexposure to pH 7.4 for 3 days. Pictures correspond to one frame per hour, starting at t=1 h. We could measure the time of first division in the presence of antibiotics at MIC levels to create figure 4D. Data from pH 5.5, as well as different antibiotic concentrations (0, 1, 10 MIC) are displayed in supplementary movie.
