# Supplementary material for: Prolonged bacterial lag time results in small colony variants that represent a sub-population of persisters
Source: Nat Commun. 2018 Oct 4;9:4074. doi: 10.1038/s41467-018-06527-0 (PMC6172231; doi:10.1038/s41467-018-06527-0)
Supplement: Supplementary file 3 — Description of Supplementary Files [file 41467_2018_6527_MOESM3_ESM.pdf]

Description of Additional Supplementary Files

File Name: Supplementary Movie 1

Description: The supplementary movie shows single bacteria cells under the microscope when they resume growth after pre-exposure to pH 5.5 and 7.4 media. Representative bacteria are shown in presence of different antibiotic concentrations corresponding to figure 4F. Even with antibiotic concentrations above MIC, bacteria manage to perform one or a few divisions before lysis.
